# The Impact of Organizational Climate Toward Sexual Minority Workers on Burnout: The Mediating Role of Quality of Work Life

**DOI:** 10.1177/10482911261475988

**Published:** 2026-08-07

**Authors:** Marco Minhós, Vítor Costa, Samuel Monteiro

**Affiliations:** 1 Departamento de Psicologia e Educação, 56056 Universidade da Beira Interior, Covilhã, Portugal; 2 CICPSI, Faculdade de Psicologia, Universidade de Lisboa, Lisboa, Portugal; 3 NECE - Research Center for Business Sciences, University of Beira Interior, Covilhã, Portugal

**Keywords:** organizational climate, sexual minorities, quality of work life, well-being, burnout

## Abstract

This study aimed to describe and analyze the relationships between the organizational climate towards sexual minority workers, the quality of work life (QoWL), and burnout in a sample of 256 Portuguese and Brazilian workers. The mediating role of QoWL in the relationship between climate and burnout was analyzed, and the variables under study were compared considering participants’ sexual orientation. Our findings showed group differences in burnout and in specific QoWL dimensions (general well-being and working conditions), but not in overall QoWL or perceived climate according to sexual orientation. An inclusive climate toward sexual minority workers had a negative impact, reducing burnout, and a positive impact on QoWL. QoWL had a negative impact on burnout and mediated the relationship between climate and burnout. We drew implications for research, practice, and policies from the current findings.

## Introduction

Sexual minority^1^ workers face disproportionate barriers and discrimination in the workplace^1^ and are at greater risk of unfair treatment, oppression, or even violence.^2,3^ When compared to their heterosexual colleagues, workers belonging to sexual minorities report higher levels of discrimination and harassment in their workplaces.^4–6^ These factors are related to poorer mental health^2,7^ and negatively impact work-family results, job satisfaction, and career decision-making.^3^ These negative interactions in the workplace can also lead to physical, psychological, and social problems, including burnout^4^ and are likely to affect workers’ quality of work life (QoWL).

Researchers have studied workplace diversity, that is, the differences in demographic and psychological characteristics found in a workforce,^8^ in the past decades. In fact, diversity training programs can be traced back to the 1970s,^8^ and models for workplace diversity can be found in the 1990s.^9^ Nonetheless, at the time, these diversity challenges were mostly limited to readily detected attributes such as sex, age, race, and ethnicity.^10^ It is only after 1990 that research starts to focus on changing institutions and promoting a positive workplace climate^11^ for sexual minorities. Still, this group appears to be under-researched when compared to other diversity groups.^12^

Organizational climate, a construct that has received over 6 decades of research attention, can be defined as the sum of processes and activities perceived by employees—including policies, procedures, and practices, as collectively perceived by staff.^13,14^ This shared perception of the climate can significantly impact various aspects of the workplace, such as recruitment, integration, commitment, stress,^15^ but also employees’ productivity,^15,16^ and mental health.^17^

Within this context, the experiences of sexual minorities in the workplace warrant special attention because they make up a significant portion of the workforce^2^ and continue to face various forms of violence and discrimination in the workplace. For example, a report made by the UNI Global Union states that around 68% of their inquiries from 51 different countries reported seeing, hearing, or experiencing any form of discrimination, harassment, or violence toward sexual minority workers.^18^ Empirical research has shown that exposure to prejudice and harassment in the workplace decreases job satisfaction,^7,19^ while a climate that supports sexual minority workers is associated with greater job satisfaction^20^ among these workers. This type of climate refers to the degree to which an organization provides instrumental and psychosocial support^21^ to this community, creating an inclusive environment,^15^ and demonstrating a general atmosphere of acceptance of one's sexual identity.^21^ Gay and lesbian workers’ perception of a more supportive climate was related to lower levels of anxiety in more demanding work environments.^22^ Additionally, it fosters stronger relationships at work, a higher level of professional commitment, and increased job satisfaction among sexual minority members.^23^ Conversely, a lack of inclusivity or a discriminatory climate can lead to significant adverse effects, not only on sexual minority employees but also on the broader workplace environment. Eckes and McCarthy^24^ report that a climate that discriminates against sexual minority teachers, besides causing harm to them, was harmful to students who witness discriminatory behavior. Eliason et al^25^ work showed the existence of a discriminatory climate toward sexual minority people in health institutions, manifested through policies that exclude staff and patients belonging to sexual minorities, derogatory comments, and attitudes that affect the professional activity of sexual minority doctors (eg, lack of referrals by heterosexual colleagues). Research also demonstrated that working in environments that lack an inclusive organizational climate for sexual minorities has a detrimental impact on heterosexual employees, harming their well-being and creating negative attitudes at work.^26^

QoWL is the extent to which an employee is satisfied with their personal and professional needs satisfaction through their participation in their workplace while trying to achieve the objectives set by the organization in which they work.^27^ It can be affected by job satisfaction and career opportunities, general feelings of happiness and life satisfaction, work-life balance, perceived pressure and/or excessive stress in the workplace, feelings of control over decisions at work, and the work environment itself.^28,29^

Regarding burnout, it has recently been conceptualized as “a work-related state of exhaustion that occurs among employees, which is characterized by extreme tiredness, reduced ability to regulate cognitive and emotional processes, and mental distancing” (p. 28).^30^ According to Schaufeli et al,^30^ these dimensions of burnout are accompanied by a depressed mood and non-specific symptoms of psychological distress and psychosomatic complaints. Burnout has been associated with (1) increased absenteeism and turnover intentions^31^; (2) reductions in productivity, job satisfaction, and commitment to the organization^31,32^; (3) lower effectiveness at work; and (4) negative effects on workers’ mental health, such as anxiety, lower self-esteem, and depression.^31^

### Organizational Climate and Burnout

There is a negative correlation between organizational climate and burnout, according to the general literature, which does not specifically address employees’ sexual orientation or identity.^33–35^ Even though there are few studies investigating the specific impact of an organization's climate toward sexual minorities on burnout, there is evidence that discriminatory and invalidating experiences seem to contribute to exhaustion, deterioration of mental health status, and the general degradation of psychological safety in the workspace among members of this community,^36^ besides exacerbating stressful situations in the workplace.^3^ According to Singh and O’Brien,^4^ greater incivility and a less inclusive climate for sexual minorities are associated with increased burnout among sexual minority employees. Rabelo and Cortina^31^ reported that sexual minority workers experiencing high levels of harassment at the workplace have higher levels of burnout (both exhaustion and disengagement), and lower levels of motivation and identification with their work.

### Organizational Climate and Quality of Work Life

Several studies have explored the association between the organizational climate experienced by workers and their QoWL^37,38^ or related dimensions.^39,40^ Studies with no focus on sexual or gender minorities, such as Itzkovich et al,^37^ found that a climate of incivility, which can include hostile looks from supervisors and coworkers, was negatively related to the quality of work-life. In turn, a supportive work environment promotes happiness at work and job satisfaction.^40^

Among sexual minority workers, Allan et al^39^ report that a climate perceived as supportive predicted greater job satisfaction, which in turn promoted life satisfaction. Among gay men, support available from the organization and perception of fair treatment were associated with higher commitment and career satisfaction.^41^

Since sexual minority employees are more likely to work in heteronormative environments characterized by discriminatory climates,^26^ they are often reluctant to disclose their sexual orientation in the workplace.^42–44^ Such concealment has been associated with detrimental outcomes such as reduced collective self-esteem, job satisfaction, and commitment.^45^ Together, a supportive workplace environment has been associated with positive work attitudes.^2^

### Quality of Work Life and Burnout

A negative relationship between QoWL and burnout has been found in recent research.^46–48^ Imbalances between the individual and various aspects of work, such as control, workload, rewards, and values, can all contribute to worker burnout and, as a result, affect performance, social behavior, and personal well-being.^32^ This negative association seems to indicate that an improvement in QoWL can be a factor in reducing workers’ burnout. In fact, Permarupan et al^49^ did a study with Malaysian nurses, in which participants reported that safe and healthy working conditions, social integration at work, and the social relevance of work (elements linked to QoWL) can significantly reduce perceptions of depersonalization in these professionals. In another study with a similar sample, the findings show that the QoWL dimensions such as social integration at work, social relevance of work, and work and total life span significantly reduce emotional exhaustion.^50^ A study carried out with primary and secondary school teachers in China reported that stress at work was positively related to their levels of burnout.^51^ This same relationship has been reported by various studies with different populations.^48,52–55^ Recent research also provides evidence of work-family conflict (a dimension of QoWL) as a predictor of burnout.^51,55^ These findings highlight the importance of QoWL and its dimensions in addressing burnout symptoms.

Thus, understanding how organizational climate toward sexual minorities affects QoWL and health outcomes, namely burnout, is a relevant and timely research topic. The current study aims to describe and analyze the relationships between the organizational climate toward sexual minority workers, QoWL, and burnout, exploring the intervening role of QoWL in the climate-burnout relationship.

## Objectives and Hypotheses

Despite the relationship found in the literature between organizational climate and burnout, there are only a small number of studies that try to address how the organizational climate toward sexual minority workers can impact burnout levels. Considering this research gap and the strong association between QoWL and burnout, the present study main objectives are: (1) to analyze the relationship between Organizational Climate toward sexual minority workers and Burnout Syndrome; (2) to analyze the relationship between Organizational Climate toward sexual minority workers and QoWL; (3) to analyze the relationship between QoWL and Burnout Syndrome; (4) to analyze the mediating role of QoWL in the relationship between Organizational Climate toward sexual minority workers and Burnout Syndrome; and (5) to compare Burnout levels, perception of QoWL and Organizational Climate for sexual minority workers by sexual orientation.

Considering the literature review, the following hypotheses were formulated:

H_1_: An inclusive Organizational Climate toward sexual minority workers has a negative impact on Burnout;
H_2_: An inclusive Organizational Climate toward sexual minority workers has a positive impact on QoWL;
H_3_: Increased QoWL has a negative impact on Burnout;
H_4_: QoWL mediates the relationship between Organizational Climate toward sexual minority workers and Burnout.

Figure 1 outlines the proposed mediation model.

**Figure 1.**
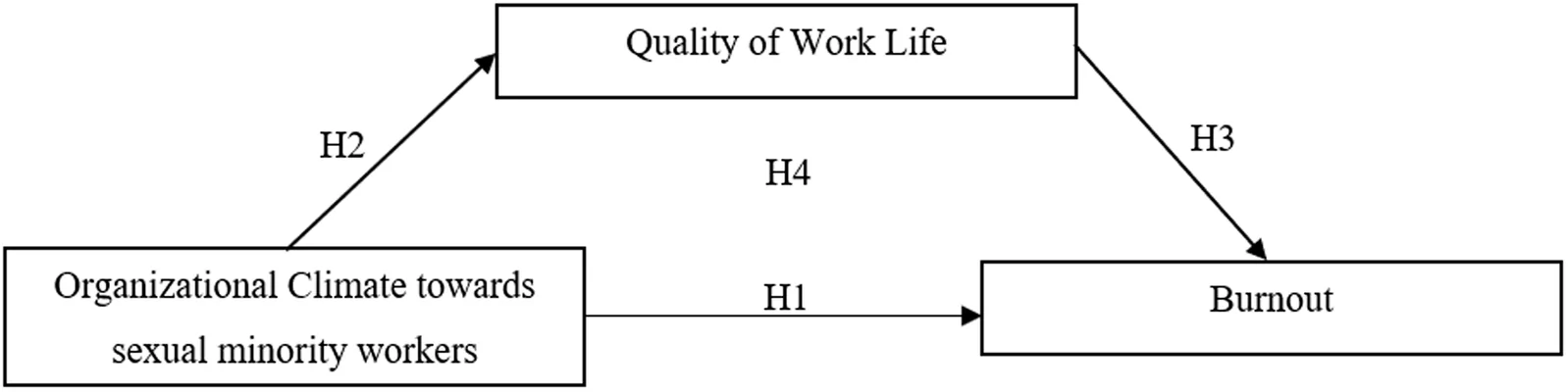
Mediation model with QoWL as mediator between organizational climate toward sexual minority workers and burnout.

## Method

### Recruitment of Participants

This study analyzed data collected as part of the Hephaestus project—“Occupational Health, Psychosocial Risks and Preventive Factors in Organizations” (coordinated by Professor Henrique Pereira), which administered an online questionnaire, between October 2020 and January 2021 (*N* = 1667). Participation in the study was voluntary. Invitations to join the study were sent by e-mail to authors’ contacts and publicly available e-mail addresses that were collected, and the study was disseminated on social media platforms (eg, LinkedIn). Materials that were used to recruit the participants contained a link to the survey website, where all relevant information was available (research objectives, filling instructions, researchers’ contacts, and the informed consent form). Since the authors were interested in collecting data among workers belonging to sexual minorities, in addition to the convenience sampling described, a purposive sampling strategy was also implemented, and thus specific groups were targeted to gather these participants for the study. The participants in this study were recruited using non-probabilistic sampling methods, which are often necessary for research involving socially invisible populations (ie, those that are difficult to reach, such as sexual minority participants, particularly in work and organizational contexts). In fact, considering the main research topic and target participants, there is no predefined population structure from which to draw and calculate a potential random sample, so probability-based sampling is not methodologically feasible in this context.^56,57^ In this specific organizational setting, non-probabilistic participant recruitment is a pragmatic and ethically appropriate strategy for reaching marginalized groups that are often underrepresented in conventional population surveys. Unfortunately, the infeasibility of probabilistic sampling does not change mathematical limitations on the interpretability of results.

From the total number of responses (*N* = 1667) we eliminated (1) participants under the age of 18 whose other related demographic information could not confirm their majority; (2) workers or working students who reported a workload less than 10 h per week; (3) students, and unemployed participants; (4) participants whose sexual orientation was unknown, and heterosexual participants who did not provide information on gender; (5) participants that submitted the survey without completing 1 or more of the study measures. Ethical principles were respected, and the study was approved by the Ethics Committee of the University of Beira Interior (n.° CEUBI-Pj-2020-088). Before starting to fill out the questionnaire, the participants had to accept the informed consent form.

### Measures

*Sociodemographic Questionnaire.* We measured gender using the questions “What is your gender? Response options were “woman,” “man,” and “other,” allowing participants to enter another gender identity. Regarding sexual orientation, we asked participants, “How do you identify in terms of sexual orientation?,” with the response options “heterosexual,” “homosexual,” and “bisexual,” and a possibility to write in another sexual orientation.

We measured burnout using the *Burnout Assessment Tool (BAT),* developed by Schaufeli et al.^30^ Composed of 22 items assessed on a Likert scale ranging from 1 (never) to 5 (always), the aim of the BAT is to assess 4 core symptoms of burnout: exhaustion (EX) which is associated with a severe loss of energy leading to a feeling of physical and mental exhaustion; emotional impairment (EI) which manifests itself through severe emotional reactions and the feeling of being overwhelmed by emotions; cognitive impairment (CI) which manifests itself in poor cognitive performance, memory problems and attention and concentration deficits; and mental distance (MD) characterized by a strong reluctance or aversion to work. By adding up the averages of these 4 core symptoms, it is also possible to ascertain the total burnout score.^30^ In this study, an internal consistency (α) of .91 was obtained for the EX scale, .89 for the EI, .85 for the CI, and .86 for the MD scale. The internal consistency of the total scale (22 items) was .95.

*Work-Related Quality of Life Scale (WRQoLS).* QoWL was assessed using the WRQoLS.^28^ This instrument consists of 23 items and an additional item that is used to measure the validity and reliability of the remaining items. The items are answered using a Likert scale ranging from 1 (strongly disagree) to 5 (strongly agree) and are made up of 6 sub-scales. General Well-Being (GWB), which assesses general feelings of happiness and satisfaction with life; the Home–Work Interface (HWI), which addresses issues related to the balance between professional and personal life; Job and Career Satisfaction (JCS), which addresses issues related to job and career satisfaction; Control at Work (CAW), which illustrates the level at which a worker feels they can have an appropriate level of control in their working environment; Working Conditions (WCS), which assesses satisfaction with working conditions, safety, and available resources; and Stress at Work (SAW), which assesses the individual's perceptions of excessive pressure and/or extreme levels of stress at work. It should be noted that the items in the SAW subscale are inverted, so the interpretation of the subscale should take this into account (ie, a higher score means less stress in the workplace). By adding up the averages of each of the subscales, it is possible to obtain the overall QoWL score.^28^ In the present study, internal consistency was .91 for GWB, .80 for HWI, .83 for JCS, .70 for CAW, .83 for WCS, and .80 for SAW. The overall internal consistency for the 23 items was .94.

*Lesbian, Gay, Bisexual, and Transgendered Climate Inventory (LGBTCI).* The organizational climate toward sexual minority workers was assessed using the LGBTCI.^15^ This instrument consists of 20 questions that measure formal and informal aspects of the organizational climate using a Likert scale from 1 (doesn’t describe at all) to 4 (describes extremely well). The overall score varies between 20 and 80 points,^15^ with higher scores indicating a more inclusive organizational climate toward sexual minority workers.^42^ The internal consistency of the scale was .95, and the average score of the scale in our sample was 57.8.

### Data Preparation and Analysis Procedures

A total of 1034 participants met the criteria for inclusion in this analysis. Data were entered into IBM Statistics SPSS, version 28. We selected all participants belonging to sexual minorities (*n* = 109) from the 1034 eligible participants for this study. Considering our aim to compare the study variables among heterosexual and sexual minority workers and knowing that “unequal sample sizes among groups influence the results” (p. 30) in statistical tests analyzing different groups,^58^ and considering the key role played by sample size and equal versus unequal sample size in statistical power,^58,59^ we randomly selected a subsample of 147 heterosexual participants (57.4% of the study sample) from the existing database using the SPSS “Select Cases” function. This approach preserves the variability of the larger group while reducing the influence of unequal variances and enhancing the comparability between groups. It also reduces the risk of overrepresenting the majority group, which could bias the results or make them less sensitive to differences between groups. Mean substitution was used to replace missing values for each individual item of the study variables (climate, QoWL, and burnout), and thus all statistics regarding the study variables were performed considering a total sample of 256 participants. Descriptive statistics (mean, median, standard deviation, minimum, maximum, skewness, and kurtosis) were calculated, and correlations were done using Spearman's rho, to include ordinal variables (eg, educational attainment, organization size). For the purposes of comparing groups according to sexual orientation, a dichotomous variable was created to distinguish between heterosexual and sexual minority participants. Differences between groups were calculated using the Mann–Whitney U test. Considering existing recommendations in the literature,^60^ we chose to consider the 1-sided *P*-value in the case of burnout and QoWL and the 2-sided *P*-value in the case of organizational climate toward sexual minority workers. To test the research hypothesis, partial least squares structural equation modeling (PLS-SEM) was used. PLS-SEM is a nonparametric statistical method, therefore appropriate for nonnormal data as it makes no assumptions regarding data distribution.^61^ However, it still assumes probabilistic sampling, a condition that could not be met for reasons described above. All analyses were done with R,^62^ version 4.5.2, with the SEMinR package,^63^ version 2.4.0.

We used the disjoint 2-stage approach,^64^ following guidelines on the implementation of higher-order constructs in PLS-SEM.^61,65^ The disjoint 2-stage approach was implemented as follows: in the first stage, the lower-order components (ie, the dimensions of burnout and QoWL) were considered in our model, linking climate to the QoWL and burnout dimensions, and the QoWL dimensions to the burnout dimensions. Then, construct scores of the burnout and QoWL dimensions were saved and added to the database. In the second stage, these scores were used to measure the higher-order constructs (ie, burnout and QoWL).

To test H1, a simple model (without the mediator) was created, considering the structural path from climate to burnout. To test H2, H3, and H4, a mediation model was created, considering the paths from climate to QoWL and burnout, and the path from QoWL to burnout.

## Results

### Participants

Our sample consisted of 256 workers, aged between 20 and 70 (*M* = 38.9; *SD* = 11.3). The average time worked at present employment was 122.8 months (*SD* = 125.3), with an average weekly workload of 38.9 h (*SD* = 13.5). These participants work mostly (88.7%) in the service sector (ie, tertiary). Table 1 shows the main sociodemographic characteristics of the sample.

**Table 1.** Sociodemographic Characteristics of the Sample (*N* = 256).

		Frequency (*n*)	Valid Percentage (%)
Gender	Man	110	43.8
	Woman	141	56.2
Sexual Orientation	Heterosexual	147	57.4
	Homosexual	64	25
	Bisexual	37	14.5
	Pansexual	6	2.3
	Asexual	2	.8
Nationality	Portuguese	156	61.2
	Brazilian	96	37.6
	Other	3	1.2
Country Where the Person Lives	Portugal	164	64.1
	Brazil	92	35.9
Marital Status	Single	115	44.9
	Married	72	28.1
	*de facto* union	49	19.1
	Divorced or separated	19	7.4
	Widowed	1	.4
Educational Attainment	Up to 9 years of schooling	7	2.7
	Up to 12 years of schooling	34	13.3
	Bachelor's degree	76	29.7
	Postgraduate or master's degree	88	34.4
	Doctorate	46	18
	Other	5	2
Place of Residence	Small rural area	13	5.1
	Large rural area	21	8.2
	Small city	106	41.4
	Large city	113	44.1
	Other	3	1.2
Socioeconomic Status	Low	9	3.5
	Low–medium	57	22.3
	Medium	153	59.8
	Medium–high	33	12.9
	High	4	1.6
Professional Status	Student worker	43	16.8
	Self-employed	33	12.9
	Employee	176	68.8
	Other	4	1.6
Nature of the Organization	Public	104	40.6
	Private	145	56.6
	Public and Private	7	2.7
Economic Sector	Primary	6	2.3
	Secondary	23	9
	Tertiary	227	88.7
Organization Size	Up to 10 people	55	21.6
	11 to 250 people	80	31.4
	251 to 500 people	25	9.8
	More than 501 people	95	37.3

### Descriptive, Correlational, and Comparative Results

In each case in which a value was missing, the mean value was substituted. In no case were more than 3.5% of the values of a variable missing. The mean values obtained in the BAT (*M* = 2.5; *SD* = .6), WRQoLS (*M* = 3.3; *SD* = .7) and LGBTCI (*M* = 2.9; *SD* = .7), when compared with the values obtained in other Western samples, show that our sample displays considerably higher levels of burnout symptoms,^30^ a slightly lower perception of QoWL,^28^ and a less inclusive perception of the climate toward sexual minority workers.^4,66^

Regarding the correlations among the variables, we found that burnout has a negative correlation with both QoWL (*r* = −.60, *P* < .01) and organizational climate toward sexual minority workers (*r* = −.15, *P* < .05). It was also observed that the organizational climate toward sexual minority workers has a positive correlation with QoWL (*r* = .34, *P* < .01). Table 2 shows the data in detail.

**Table 2.** Correlations Between the Study Variables.

	M	SD	Skew	Kurt	1	2	3	4	5	6	7	8	9	10	11	12	13	14	15	16	17	18	19
1-AGE	38.9	11.3	.290	−.826	—																		
2-EA	−	−	−	−	.18**	—																	
3-SES	−	−	−	−	.27**	.30**	—																
4-PE	122.6	125.3	1.128	.409	.71**	.24**	.24**	—															
5-WW	38.3	10.3	.134	2.998	.02	−.02	−.10	.05	—														
6-O_Size	−	−	−	−	.31**	.32**	.32**	.17**	−.07	—													
7-LGBT Climate	2.9	0.7	−.355	−.113	−.10	−.08	.01	−.07	−.02	−.15*	—												
8-CAW	3.5	0.9	−.495	−.040	.06	.06	.20**	.11	.01	−.03	.22**	—											
9-JCS	3.5	0.8	−.561	.100	.06	−.04	.23**	.12	−.05	.02	.30**	.59**	—										
10-GWB	3.3	0.9	−.448	−.320	.09	−.06	.22**	.09	−.07	.10	.18**	.36**	.68**	—									
11-HWI	3.4	1.0	−.483	−.252	.02	−.08	.11	.08	−.13*	.03	.31**	.33**	.53**	.51**	—								
12-WCS	3.4	1.0	−.552	−.337	−.08	−.22**	.12*	−.04	−.16**	−.11	.34**	.33**	.60**	.53**	.73**	—							
13-SAW	2.8	1.1	.059	−.799	.02	−.06	.08	−.08	−.17**	−.06	.19**	.03	.28**	.34**	.41**	.43**	—						
14-QoWL	3.3	0.7	−.594	.513	.04	−.11	.20**	.06	−.13*	−.02	.34**	.53**	.79**	.75**	.81**	.83**	.60**	—					
15-EX	2.8	0.7	.412	.335	−.13*	.00	−.13*	.00	.09	−.07	−.10	−.22**	−.44**	−.56**	−.46**	−.45**	−.54**	−.62**	—				
16-MD	2.0	0.8	1.0	0.7	−.10	−.03	−.12*	−.08	−.02	.04	−.23**	−.29**	−.59**	−.53**	−.41**	−.41**	−.37**	−.58**	.59**	—			
17-CI	2.4	0.7	.688	1.048	−.21**	.05	−.14*	−.12*	.00	.00	−.14*	−.26**	−.42**	−.47**	−.35**	−.32**	−.28**	−.46**	.57**	.56**	—		
18-EI	2.5	0.8	.576	.237	−.08	.05	−.15*	−.03	−.03	−.06	−.12	−.12	−.33**	−.47**	−.32**	−.29**	−.30**	−.40**	.57**	.47**	.62**	—	
19-Burnout	2.5	0.6	.711	.797	−.17**	.01	−.17**	−.08	.06	−.07	−.15*	−.24**	−.49**	−.59**	−.44**	−.41**	−.47**	−.60**	.85**	.75**	.78**	.78**	—

*Note.* EA = educational attainment; SES = socioeconomic status; PE = professional experience; WW = weekly workload; O_Size = organization size; EX = exhaustion; MD = mental distance; CI = cognitive impairment; EI = emotional impairment; QoWL = Quality of Work Life; CAW = Control at Work; JCS = Job and Career Satisfaction; GWB = General Well-Being; HWI = Home–Work Interface; WCS = Working Conditions; SAW = Stress at Work.

*P < .05; **P < .01

As for the comparison between groups, differences were found in burnout (overall) and all subscales (except mental distance), with sexual minority participants showing higher levels (mean rank). In what concerns quality of working life, differences were only found in the general well-being and working conditions dimensions, with heterosexual participants’ QoWL being significantly different (better) in these dimensions. Little difference was found in the LGBT climate. The results can be seen in Table 3.

**Table 3.** Group Differences by Sexual Orientation in Burnout, QoWL, and LGBT Climate.

	Heterosexual (*n* = 147)	Sexual Minority (*n* = 109)	
	Mean Rank	Mean Rank	*z*-Value
BAT	116.82	144.25	−2.93*
EX	121.34	138.16	−1.80*
MD	122.02	137.24	−1.64
CI	121.69	137.69	−1.72*
EI	116.57	144.58	−3.00*
WRQoLS	134.88	119.89	−1.60
CAW	129.16	127.61	−0.17
JSC	134.52	120.39	−1.51
GWB	139.11	114.20	−2.67*
HWI	131.76	124.11	−0.82
WCS	135.43	119.16	−1.75*
SAW	131.36	124.64	−0.73
LGBTCI	133.68	121.52	−1.30

*Note*. BAT = *Burnout Assessment Tool*; EX = exhaustion; MD = mental distance; CI = cognitive impairment; EI = emotional impairment; WRQoLS = Work-Related Quality of Life Scale; CAW = Control at Work; JCS = Job and Career Satisfaction; GWB = General Well-Being; HWI = Home-Work Interface; WCS = Working Conditions; SAW = Stress at Work; LGBTCI = Lesbian, Gay, Bisexual and Transgendered Climate Inventory

*P < .05, 1-sided

### Measurement Model, Structural Model, and Hypotheses Testing

At stage 1, all dimensions of burnout and QoWL showed good internal consistency (Cronbach alpha values were all >.70), internal consistency reliability (rhoA and rhoC values were all > .70), and convergent validity (average variance extracted (AVE) values >.50). Concerning stage 2, the measurement model is presented in Table 4. As can be seen, measures are reliable, and convergent validity is achieved. While some outer loadings are below the ideal value (.708), since the threshold of internal consistency and reliability values is achieved, we retained the indicator following Hair et al's (2017) recommendation of keeping indicators with outer loading between .40 and .70. Discriminant validity between the constructs is achieved, with the maximum values for the heterotrait–monotrait (HTMT) ratio of correlations^67^ being .78, which is below the more restrictive threshold of .85.

**Table 4.** Measurement Model.

Constructs	Items^a^	Outer Loadings	Cronbach Alpha	rhoA	AVE
Burnout	EX	.875	.86	.88	.70
	MD	.845			
	CI	.844			
	EI	.785			
Quality of Work Life	JCS	.884	.86	.88	.59
	CAW	.616			
	GWB	.818			
	HWI	.829			
	SAW	.572			
	WCS	.849			
LGBT Climate	LGBTCI_1	.816	.95	.96	.51
	LGBTCI_2	.587			
	LGBTCI_3	.737			
	LGBTCI_4	.812			
	LGBTCI_5	.745			
	LGBTCI_6	.648			
	LGBTCI_7	.790			
	LGBTCI_8	.594			
	LGBTCI_9	.658			
	LGBTCI_10	.598			
	LGBTCI_11	.727			
	LGBTCI_12	.756			
	LGBTCI_13	.728			
	LGBTCI_14	.761			
	LGBTCI_15	.656			
	LGBTCI_16	.746			
	LGBTCI_17	.855			
	LGBTCI_18	.452			
	LGBTCI_19	.778			
	LGBTCI_20	.779			

*Note*: AVE = average variance extracted.

a Burnout and QoWL items are latent variable scores from stage 1 of the disjoint 2-stage approach.

Regarding the test of research hypotheses, a path coefficient of −.32 (95%CI [−.44, −.24]) was obtained, and therefore our sample does not provide evidence for rejection of H_1._ In our sample, climate toward sexual minority workers explains 10% of the variance of burnout. The results of the mediation model can be seen in Table 5. In our sample, the organizational climate toward sexual minority workers has a positive impact on QoWL with a path coefficient of .43 (95%CI [.32,.55]). In turn, QoWL negatively predicts burnout with a coefficient of −.69 (95%CI [−.77,−.62]). Therefore, our sample does not provide evidence for rejection of either H_2_ or H_3._ It was also found that the indirect effect of the organizational climate toward sexual minority workers on burnout, through QoWL, was −.30 (95%CI [ −.40, −.22]), and thus our sample did not provide evidence for rejection of H_4._ When QoWL is considered in our model as a mediator, the path coefficient from LGBT climate to burnout was close to zero (.01), with confidence intervals including zero (95%CI [−.10, .12]), suggesting that the effect of organizational climate on burnout was mediated by QoWL. *R*^2^ values were obtained, showing that 19% of QoWL variance is explained by LGBT climate, while 48% of burnout's variance is explained by both LGBT climate and QoWL. Effect size (*f*^2^ values) was also calculated, showing a medium effect of LGBT climate on QoWL (*f*^2^ = .23), and a large effect of QoWL on burnout (*f*^2^ = .73). The effect of LGBT climate on burnout was less than .02, indicating no effect.^68^ Since our study included 256 participants, it meets the commonly recommended thresholds for obtaining reliable estimates of variance explained (*R*^2^). According to methodological guidelines, sample sizes of at least 200 to 250 participants are generally required to ensure stable and unbiased *R*^2^ values in regression and structural equation modeling.^58,69^ According to Hair et al*,*^58^ with a power of .80, and a sample size of *N* = 250, given 2 independent variables (as is the case in our study), the minimum *R*^2^ that can be found statistically significant is *R*^2^ = 4 (cf. Hair et al*,*^58^ p. 279, Figure 5.8). Therefore, reporting that 48% of the variance in burnout was explained by climate toward sexual minority workers and QoWL can be considered a valid and robust estimate, given our sample size and considering 2 independent variables. However, due to the non-probabilistic nature of the sample, the generalizability of the results beyond the sample is unclear.

**Table 5.** Direct, Indirect, and Total Effects.


Path	Path Coeff.	Boot. M	Boot. SD	*T*-value	95%CI	Boot. *P-*value
*Direct Effects*						
LGBTCI to QoWL	.43	.44	.06	7.29	[.32, .55]	0.00
LGBTCI to BAT	.01	.01	.06	0.13	[−.10, .12]	0.90
QoWL to BAT	−.69	−.70	.04	−18.64	[−.77, −.62]	0.00
*Indirect Effects*						
LGBTCI to BAT	−.30	−.31	.05	−6.60	[−.40, −.22]	0.00
*Total Effects*						
LGBTCI to QoWL	.43	.44	.06	7.29	[.32,.55]	0.00
LGBTCI to BAT	−.29	−.30	.07	−4.52	[−.42, −.17]	0.00
QoWL to BAT	−.69	−.70	.04	−18.64	[−.77, −.62]	0.00

## Discussion

In our sample, sexual minority participants reported higher burnout compared to heterosexual participants. However, their perception of QoWL and organizational climate toward sexual minorities was not much different from that of heterosexual participants. More specifically, sexual minority participants reported higher exhaustion in the workplace, decreased interest and enthusiasm for their work, poorer cognitive performance, more difficulties dealing with emotions, greater dissatisfaction with working conditions, and poorer general well-being. These findings partially support previous research indicating that sexual minority workers perceive a more negative QoWL and health than heterosexual workers.^2,4,7^

Secondly, this study provides evidence that an inclusive climate toward sexual minority workers has a negative impact on burnout, meaning that the more inclusive the climate, the lower the levels of burnout among all workers. This appears to be 1 of the first studies to investigate the predictive value of this type of climate on burnout symptoms in workers. However, previous studies^4,19^ have already shown a significant negative correlation between this type of climate and burnout, as well as the fact that the absence of an inclusive organizational climate for sexual minorities was associated with lower well-being of all workers.^26^

The results also indicated that a climate that is more supportive of sexual minority workers has a positive impact on QoWL, meaning that the more inclusive the climate, the greater the perception of QoWL among all workers. This result is in line with previous studies, which found positive associations between a supportive organizational climate and both job satisfaction and subjective well-being dimensions such as life satisfaction.^39,70^

In line with previous research,^70–72^ our results showed a negative impact of QoWL on burnout, which tells us that the higher the perception of QoWL, the lower the burnout levels of the workers tend to be.

The mediation analysis, conducted across the full sample (heterosexual and sexual minority participants), revealed that QoWL mediates the relationship between organizational climate and burnout. This suggests that employees’ perception of a supportive workplace climate toward sexual minority workers contributes to employees’ well-being primarily by enhancing QoWL, which in turn reduces burnout symptoms.

Following this analysis and considering how our sample mean values in the LGBTCI, WRQoLS, and BAT, compare with other studies,^4,28,30,66^ we provide evidence for promoting a climate of greater support for sexual minority workers, which may in turn achieve an improvement in the QoWL of Portuguese and Brazilian employees and potentially reduce burnout. This conclusion is especially important for the sexual minority workers in this sample, given that they reported worse perceived QoWL and higher levels of burnout compared to heterosexual participants. It therefore seems crucial to promote an inclusive organizational climate toward sexual minority workers, so that this inclusive climate can contribute to a reduction in harm/promotion of workers’ health.

The present study shows that an inclusive organizational climate was associated with both job satisfaction and well-being (increasing QoWL) and with workers’ health (reducing burnout). In turn, QoWL reduced burnout directly and indirectly by mediating the effect of climate. The findings provide evidence for the importance of understanding the relationships between these variables (ie, climate, QoWL, and burnout) in promoting well-being and health in various workplace settings. In particular, we underline the fact that an inclusive organizational climate toward sexual minority workers—similarly perceived by both heterosexual and sexual minority workers from our sample—is associated with lower burnout and higher QoWL for these workers, therefore benefiting not only sexual minorities but also heterosexual workers.

Our findings have several implications for research, practice, and policies. In terms of research, this study contributes to the literature by extending research on the mechanisms through which workplace climate influences burnout, providing empirical evidence for the importance of an inclusive organizational climate in occupational health research, suggesting that inclusive work environments are crucial to increase employees’ perceived QoWL and reduce burnout. Given that, to our knowledge, this is the first study to test this mediation model, it is critical to replicate it on working populations from various sectors of the economy, particularly since the tertiary sector (ie, services) is overrepresented in this sample. It may also be important to analyze the moderating effect of sexual orientation on the relationships tested in this model to understand to what extent the results observed depend on the sexual orientation of the participants.

In terms of implications for practitioners and policymakers, these findings emphasize the importance of fostering an inclusive workplace climate to improve employees’ QoWL and reduce burnout, which could help workplaces structure their diversity programs to promote inclusivity and psychological safety for sexual minority employees. Therefore, the current research contributes with relevant results that may have policy implications for the legislative efforts in place to promote workplace inclusivity in Portugal and Brazil, as has already been done in other social domains.^73^ In Portugal, the National Strategy for Equality and Non-Discrimination (ENIND 2018–2030) and its associated action plans address discrimination based on sexual orientation and gender identity.^74^ The Portuguese Labor Code mandates employers to prevent harassment and discrimination, with enforcement mechanisms supported by the Commission for Equality in Labor and Employment, with a focus on equality and non-discrimination between men and women at work.^75^ In Brazil, the Federal Constitution and Law No. 9.029/1995^76^ prohibit employment discrimination based on sexual orientation. Employers are urged by technical notes released by the Public Labor Prosecution Office (MPT) to ensure inclusive workplaces and prevent psychosocial risks.^77^ Organizations such as the Commission for Citizenship and Gender Equality (CIG) in Portugal, created in 1977,^78^ and the National LGBTQIA+ Rights Council in Brazil^79^ actively shape policies and promote inclusive labor practices. Judicial avenues and independent bodies such as labor courts, the Public Labor Prosecution Office in Brazil, and the Commission for Equality in Labor and Employment in Portugal provide employees with means to report discrimination and enforce their rights. More broadly, the legal framework considers it a general obligation of the employer to ensure that exposure to psychosocial risks does not endanger workers’ health,^80^ which may contribute to preventing the exclusion of workers in general and those belonging to minority groups in particular.

Considering the current legislative landscape in Portugal and Brazil, we recommend that: diversity and inclusion plans should be made mandatory for medium and large organizations, accompanied by publicly available annual reports documenting progress in the inclusion of sexual minority workers; workplace training requirements should be tied to public procurement eligibility, whereby companies bidding for state contracts are required to demonstrate certified inclusion training; and organizations should be required to implement an external ombudsperson mechanism for harassment complaints, ensuring that reporting channels remain independent of internal human resources structures and thereby reducing the risk of retaliation and underreporting among sexual minority employees.

At the individual level, this study has implications for any occupationally active adult, as workers’ attitudes as well as interpersonal and labor relations are key to inclusion.^81^ Our findings have implications for managers, human resource practitioners, and employees who participate in voluntary affirmative action initiatives, inclusive hiring practices, and internal reporting mechanisms. In Portugal, initiatives and strategies on diversity, equity, and inclusion can be found and are publicly shared by companies such as BNP Paribas Portugal and NOS (a telecommunications and media company).^82,83^

It is also important to note that our results indicate that inclusivity is beneficial for employees in general, which aligns with prior research suggesting that all employees, regardless of sexual orientation, can benefit from inclusive climates,^26^ adding an explanatory model of how a supportive workplace climate can contribute to employees’ well-being. However, future research should further examine the specific mechanisms through which inclusivity influences different demographic groups and whether certain employees experience greater benefits than others. Moreover, organizations should recognize that fostering an inclusive climate is an ongoing process requiring continuous assessment and adaptation to employees’ evolving needs.

This study is not without limitations. Workers who are unfamiliar with technology and/or do not have access to it may have been excluded from this study due to the (online) data collection method. This method of data collection also prevents participants from receiving immediate assistance if they are unsure how to fill out the questions. Furthermore, the fact that this is a cross-sectional study makes it impossible to establish causal relationships. It is also important to note that nonprobability sampling procedures (convenience and purposive sampling) were used, since random sampling was not feasible in this study. As a result, only nonparametric statistics were used. Therefore, our results should be interpreted as conditional on the analytic model rather than as population estimates. As a result, the findings presented in this study should be interpreted as indicative of trends within this specific group of respondents, rather than as representative estimates of the larger workforce populations in Portugal and/or Brazil. The number of participants, although relevant and appropriate to the complexity of the model tested, should be expanded in future studies to ensure that it does not interfere with a valid estimation of variance explained.

The use of self-reported measures may also introduce bias, as perceptions of organizational climate can be subjective. Future studies could incorporate objective indicators in the assessment of organizational climate, such as actual company policies and practices. Another limitation is that our study did not control for potential moderating factors such as job tenure, organizational hierarchy, or industry type. Different work environments may present varying levels of inclusivity and stress, potentially influencing the relationships observed in our study. Future research should consider these factors to provide a more comprehensive analysis of workplace inclusivity and well-being. It is also worth noting that in the mediation model, the total scores of the study variables were entered, and therefore the dimensions of QoWL and burnout were not individually considered. While this approach allowed us to investigate the overall relationships between these variables, we are unable to determine which dimensions of QoWL are more associated with organizational climate or play a greater role in burnout's dimensions. Examining these elements independently could yield more profound understandings of the ways in which sexual minority climate perceptions relate to various aspects of well-being. Despite these limitations, this study underscores the urgent need for organizations to implement strategies fostering an inclusive climate to improve employee well-being.

In conclusion, the present study provides a valuable contribution to the literature, exploring the health consequences of perceived climate toward sexual minority workers and QoWL, as well as the mediating role played by QoWL in the climate–burnout relationship.

## Notes

1. Throughout this article, we use sexual minority to refer to participants who identified as lesbian, gay, or bisexual (LGB). Although the instrument used refers to LGBT Climate, our sample did not include transgender participants; we therefore interpret results as reflecting the experiences of sexual minority (LGB) individuals specifically.
